# Acid Versus Amide—Facts and Fallacies: A Case Study in Glycomimetic Ligand Design

**DOI:** 10.3390/molecules30244751

**Published:** 2025-12-12

**Authors:** Martin Smieško, Roman P. Jakob, Tobias Mühlethaler, Roland C. Preston, Timm Maier, Beat Ernst

**Affiliations:** 1 Computational Pharmacy, Department of Pharmaceutical Sciences, University of Basel, Klingelbergstrasse 50, 4056 Basel, Switzerland; martin.smiesko@unibas.ch; 2 Department Biozentrum, Structural Area Focal Biology, University of Basel, Spitalstrasse 41, 4056 Basel, Switzerland; 3 Molecular Pharmacy, Department of Pharmaceutical Sciences, University of Basel, Klingelbergstrasse 50, 4056 Basel, Switzerland

**Keywords:** E-selectin antagonists, bioisosteres, amides as carboxylate isosteres, pre-organization, glycomimetics, quantum mechanics methods

## Abstract

The replacement of ionizable functional groups that are predominantly charged at physiological pH with neutral bioisosteres is a common strategy in medicinal chemistry; however, its impact on binding affinity is often context-dependent. Here, we investigated a series of amide derivatives of a glycomimetic E-selectin ligand, in which the carboxylate group of the lead compound is substituted with a range of amide and isosteric analogs. Despite the expected loss of the salt-bridge interaction with Arg97, several amides retained or even improved the binding affinity. Co-crystal structures revealed conserved binding poses across the series, with consistent interactions involving the carbonyl oxygen of the amide and the key residues Tyr48 and Arg97. High-level quantum chemical calculations ruled out a direct correlation between carbonyl partial charges and affinity. Instead, a moderate correlation was observed between ligand binding and the out-of-plane pyramidality of the amide nitrogen, suggesting a favorable steric adaptation within the binding site. Molecular dynamics (MD) simulations revealed that high-affinity ligands exhibit enhanced solution-phase pre-organization toward the bioactive conformation, likely reducing the entropic penalty upon binding. Further analysis of protein–ligand complexes using Molecular mechanics/Generalized born surface area (MM-GB/SA) decomposition suggested minor lipophilic contributions from amide substituents. Taken together, this work underscores the importance of geometric and conformational descriptors, beyond classical electrostatics, in driving affinity in glycomimetic ligand design and provides new insights into the nuanced role of amides as carboxylate isosteres in protein–ligand recognition.

## 1. Introduction

E-selectin, an inducible C-type lectin expressed on activated endothelial cells, plays a central role in inflammation and metastasis by mediating leukocyte rolling through interactions with the sialyl Lewis^x^ (**1**, sLe^x^) tetrasaccharide motif on leukocytes [1,2]. At the binding site of the E-selectin, the carboxyl of sLe^x^ is pivotal for its recognition by forming a salt bridge to Arg97 and by accepting an H-bond from Tyr48 [3]. As sLe^x^ (**1**) violates most of the drug-likeness rules [4,5], multiple previous studies [6,7,8,9] urged to rationally optimize sLe^x^ (**1**) to obtain high-affinity and as the ultimate goal orally available E-selectin antagonist.

In a series of rationally designed studies, we succeeded in identifying the common molecular scaffold that ensures optimal interaction of antagonists with E-selectin, which can be concluded as follows. The three hydroxyls of the fucose unit have been confirmed as the main recognition element forming the bidentate interaction from the OH groups at positions 3 and 4 to the calcium ion, which additionally engages in H-bonds with Glu80 and Glu107 and donates an H-bond from 2-OH to Glu88 [10]. In contrast, the *N*-acetylglucosamine unit of sLe^x^ (**1**) can be replaced by simpler synthetic aliphatic ring scaffolds devoid of polar atoms [11]. Because of its essential role in core pre-organization via a non-conventional H-bond between H-5^Fuc^ and O-5^Gal^ [12,13,14], the galactose unit offers little potential for optimization. Moreover, galactose hydroxyls at position 4 and 6 engage in direct H-bonding with E-selectin. Therefore, the majority of potent E-selectin antagonists are built around the unchanged galactose unit, with some exceptions having lipophilic substituents at position 2 (acetyl, benzoyl) [15] that may further support ligand structure pre-organization (through the steric effect from voluminous equatorial substituents and/or hydrophobic collapse in lactate-based ligands). The neuraminic acid unit in sLe^x^ (**1**) and especially its essential carbonyl group can be favorably mimicked by lipophilic lactic acid derivatives, most notably, cyclohexyllactic acid [16,17]. However, carboxylic acids in drug candidates often suffer from drawbacks such as high polarity and full ionization at physiological pH, limiting membrane permeability and bioavailability, and are frequently metabolized (e.g., via glucuronidation) [18,19]. While the polarity (polar surface area) of the prototypic tetrasaccharide E-selectin antagonist containing fucose and galactose units cannot be further reduced, replacement of the carboxylic acid functional group offers unique potential to improve affinity and membrane transfer.

In the search for bioisosteric replacement of carboxyl groups [20,21], non-ionic isosteres, such as amides, are particularly attractive because they retain hydrogen bonding capabilities while reducing polarity and avoiding the negative charge associated with the predominant carboxylate form at physiological pH. Crystal structures of sLe^x^ and its mimics [10] show that only one of the oxygen atoms of the carboxyl group is directly involved in a salt-bridge (Arg97) and a H-bonding (Tyr48) interactions, thus rendering the carboxyl group an ideal candidate for conversion to amide.

Amide bioisosterism is a well-established concept in drug design [22,23,24], contributing to improvements in potency, selectivity, and pharmacokinetic profiles of various classes of compounds. Conversion of a charged carboxylate into a neutral amide removes the salt bridge but maintains the potential for directional hydrogen bonds to the oxygen atom. Due to a favorable electron pair delocalization from the amide nitrogen toward carbonyl, increased charge density can be detected on the oxygen atom, making it a perfect isostere of the carboxyl group at the E-selectin binding site. Several high-resolution crystal structures of *N*,*N*-dialkyl amides deposited at the small molecule crystal structure database (Table 1) show that as many as three simultaneous H-bonds can be accepted by the amide carbonyl oxygen atom, confirming extended charge delocalization towards the oxygen atom. In addition, the absence of a formal charge in amides often improves lipophilicity, hence passive permeability and oral absorption, which are critical for systemic and oral therapies.

Another advantage of amides over a carboxylate is their synthetic and structural versatility. *N*-substituted amides enable structure–activity relationships tuning through steric and electronic modifications, which can influence binding geometry, water displacement, and internal conformational preferences. In addition, amides are more stable than acids, primarily due to their higher stabilization by resonance. With respect to metabolic stability carboxyls are much easier deactivated by glucuronidation, rendering them much less stable than amides [18,19].

Despite the undisputed theoretical benefits mentioned earlier, acid-to-amide replacements can also compromise binding, especially if the carboxylate negative charge is essential for affinity. Such replacements do not always maintain biological activity, highlighting the need for the systematic evaluation of structural and dynamic compensation mechanisms.

In this study, we investigated the acid-to-amide replacement paradigm for E-selectin glycomimetics by replacing the carboxylate group of glycomimetic **2** with a series of amide substitutions (⟶ **3a-n**) (Table 1).

We experimentally and computationally explored chemometric, structural, and dynamical trends to determine whether amides can compensate for lost ionic interactions. Based on the data collected from the crystal structures (see Table 1), we attempted to decode how the amide functionalities interact with E-selectin. From a computational perspective, we rely on molecular dynamics (MD) simulations of both free ligands and protein–ligand complexes to assess solvation patterns, conformational dynamics, and key intermolecular interactions. Through this multifaceted approach, we aimed to distinguish well-supported facts from common fallacies regarding acid-to-amide bioisosterism in glycomimetic drug design.

## 2. Results

Diverse aliphatic amide derivatives of lead compound **2** were synthesized [25] (Table 1), including the unsubstituted amide **3a**, *N*-alkylated amides **3b**&**3c**, *N*,*N*-dimethylamide **3d**, cyclic amides **3e**&**3f** and substituted cyclic amides **3g**–**3l**.

Compounds **3d**–**3g** and **3k** were co-crystallized with E-selectin, and their structures were resolved by X-ray diffraction (see Supporting Information). Isothermal titration calorimetry (ITC, see Supporting Information) was used to probe the enthalpic and entropic contributions of lead compound **2** [26] (ΔG = −24.1 kJ/mol, ΔH = −5.3 kJ/mol, −TΔS = −18.8 kJ/mol) and amide derivative **3f** (ΔG = −26.4 kJ/mol, ΔH = −6.7 kJ/mol, −TΔS = −19.7 kJ/mol). Binding free energies obtained with are in excellent agreement with values derived from affinity measurements (**2**: ΔG = −24.07 kJ/mol; **3f**: ΔG = −27.05 kJ/mol).

Despite the fact that measured binding affinity data span a rather narrow range (*K*_d_ range from 383 µM to 11.4 µM, corresponding to a ΔΔG range of 8.7 kJ/mol (2.1 kcal/mol), they offer ample room for intriguing interpretation. Compared to carboxylate lead **2**, unsubstituted amide **3a** and *N*-monoalkylated amides **3b**&**3c** showed a slight worsening of the binding affinity by a factor of 1.5, whereas *N*,*N*-dimethylamide **3d** and cyclic amides **3e**–**3l** show moderately improved affinities (up to a factor of 3.5).

### 2.1. Crystallographic Analysis

**Figure 1:**
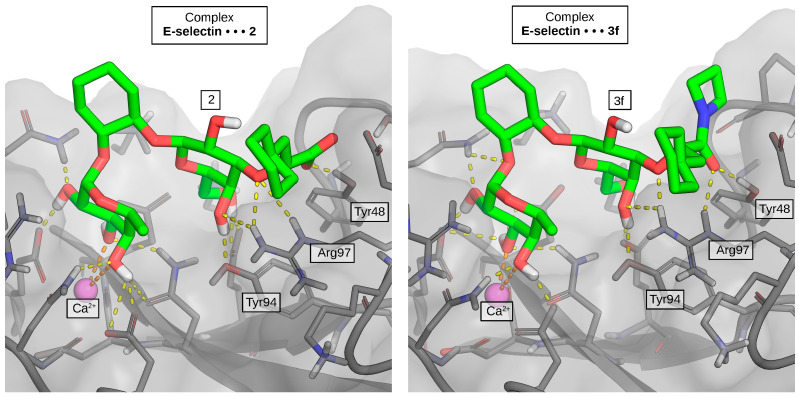
Prepared X-ray structures of the lead structure **2** (**left**) and the azetidine amide **3f** (**right**) in green sticks representation showing consistency in binding modes (calcium ion is represented by a purple sphere, protein surface semitransparent).

Crystallographic data confirmed the assumed binding modes for the amide derivatives and highlighted their compatibility in terms of isosteric and isoelectronic properties with respect to carboxylate **2** (Figure 1, Supporting Information Figure S1). In all resolved structures, the trisaccharide part mimicking the Le^x^ core (fucose and galactose linked by the cyclohexane moiety) adopts an identical, undisturbed pose, as seen in the crystal structure of lead compound **2** (PDB ID: 6EYI). Focusing on the amide moieties presumably reveals the optimal orientation of the amide carbonyl for accepting H-bonds from Arg97 and Tyr48; however, detailed visual inspection does not provide any further clues to explain the structure–activity relationships in our series. Unfortunately, the accuracy of the collected X-ray diffraction data and electron densities derived thereof does not suffice for an unambiguous interpretation of structural data, i.e., the variability in distances and angles of the key protein-ligand interactions with respect to resolution is not significant.

From a medicinal chemistry point of view, a major aspect to consider in the rational design of congeneric ligand series is the replacement of charged functional groups. As mentioned earlier, the carboxylate group of lead molecule **2** accepts H-bonds from the Tyr48 hydroxyl to the *endo* lone pair of one oxygen atom, and from the positively charged guanidinium group (Ne-H donor) of Arg97 to the *exo* lone pair of the same oxygen atom. Thus, the replacement of a carboxylate with a neutral amide may seem counterproductive because of the loss of the salt bridge.

**Figure 2:**
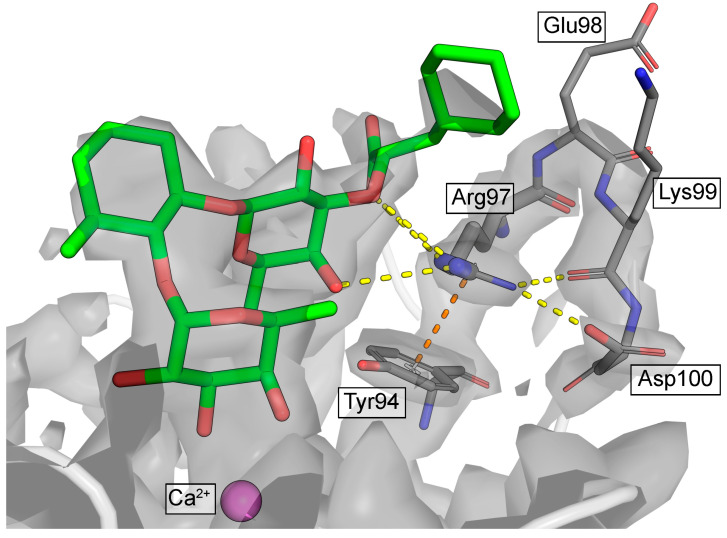
Electron density (grey contours) at the Arg97-Glu98-Lys99-Asp100 loop (PDB ID: 4C16). Yellow dashed lines indicate H-bonding/salt-bridge interactions, orange dashed line indicates the cation-pi interaction.

However, the final effect of such a replacement must be evaluated in a broader context, i.e., not only directly interacting charged residues within the range of electrostatic cutoffs. In the best resolved E-selectin/mimetic X-ray structure (PDB 4C16; resolution 1.93 Å), Arg97 is part of a loop formed by four consecutive charged residues: Arg97, Glu98, Lys99, and Asp100 (Figure 2). Residues Glu98 and Lys99 are oriented toward the bulk solvent and likely enjoy considerable conformational freedom (side chains of these residues are only partially resolved or completely smeared out). However, Arg97 and Asp100 are very well resolved, and besides H-bonds to the backbone, they form a stable salt bridge observed in all crystal structures of E-selectin. This means, that Arg97 with a permanent countercharge at its side, further supported by the co-planar cation-π interaction with the electron-rich Tyr94, may not necessarily need a further negatively charged partner from the ligand side.

### 2.2. Electrostatic Descriptors

This motivated us to explore the differences in charge-related descriptors of amides with varying substitution patterns. The amides studied consisted almost exclusively of single bonds, ruling out the possibility of far-reaching electron delocalization effects. Therefore, we simplified our model systems of tetrasaccharide mimics to a series of much smaller acetic acid isosteres (i.e., by neglecting the common ligand part, **2** was simplified to acetic acid, **3a** to *N*,*N*-dimethylacetamide, etc. (see Supporting Information, Figure S2). In turn, the smaller size and lower atom counts allowed us to employ high-level ab initio methods in the gas and solvent phases for the precise quantification of relevant descriptors. First, we analyzed the charge density of the carbonyl oxygen. Because of electron pair delocalization from the nitrogen toward the carbonyl of the amide group, a pronounced negative charge can build up on the oxygen atom, which, in our setting, should favor strong H-bonding to Arg97 and Tyr48. However, natural bond orbital analysis (NBO3.1) did not show any charge-dependent correlation (Table 2) in our series. Partial charges (i.e., electron density) at the carbonyl oxygen of the most affine amides **3f**–**3l**, featuring a rather strained azetidine amide ring with hindered electron delocalization, reach almost identical values as unstrained, ideally delocalized *N*-monoalkyl amides **3b**&**3c** and *N*,*N*-dimethyl amide **3d**, which have a weaker affinity than lead **2**. However, the resulting charge on the carbonyl oxygen is a product of multiple concurrent local effects, e.g., open or cyclic structures, bond angles, ring size, electron-donating or electron-withdrawing substituents, and bulkiness. This suggests that attempting to generalize global trends from single-atom electrostatics, despite their key importance, may not be reliable in this context.

Notably, in all azetidine amides, the nitrogen atom exhibits pronounced pyramidality (defined as the out-of-plane angle f (R_1_, R_2_, C_carbonyl_, N)), ranging from 7.1° to 18.9° (Table 2), whereas an ideally sp^3^-hybdridized nitrogen shows an out-of-plane angle of 35.3°. Surprisingly, such pyramidality seems to be moderately positively correlated (R^2^ = 0.569) with affinity, with distorted planarity favoring binding (Supporting Information, Figure S3).

As electronic effects were ruled out earlier, a potential explanation is that planar amides with a naturally wider angle between substituents a(R_1_, N, R_2_) (≈120° in *N*,*N*-dimethyl amide) might suffer from a poorer steric fit into the H-bonding cavity than amides with pyramidal nitrogen and naturally narrower a(R_1_, N, R_2_) angle (≈95° in azetidine amides). Finally, none of the additional physics-based ab initio descriptors that we further explored, including gas/solvent-phase dipole moments and desolvation energies, offered hints for a better interpretation of structure–activity relationships.

### 2.3. Conformational Pre-Organization from Molecular Dynamics (MD)

Previous studies have shown that carbohydrate mimics often exhibit complex conformational behavior, profiting from a variety of intramolecular interactions that drive their structural pre-organization. Ernst et al. [16,17] recognized that the pre-organization of the sLe^x^ tetrasaccharide can be conveniently represented by two distinct torsional parameters, termed *core conformation* and *acid orientation*. As static modeling, that is, superposition of global minima or crystal structure poses, does not provide sufficient discriminative power for such fine structural parameters, we employed MD simulations to obtain time-dependent, refined torsional parameters for isolated ligands in water.

**Figure 3:**
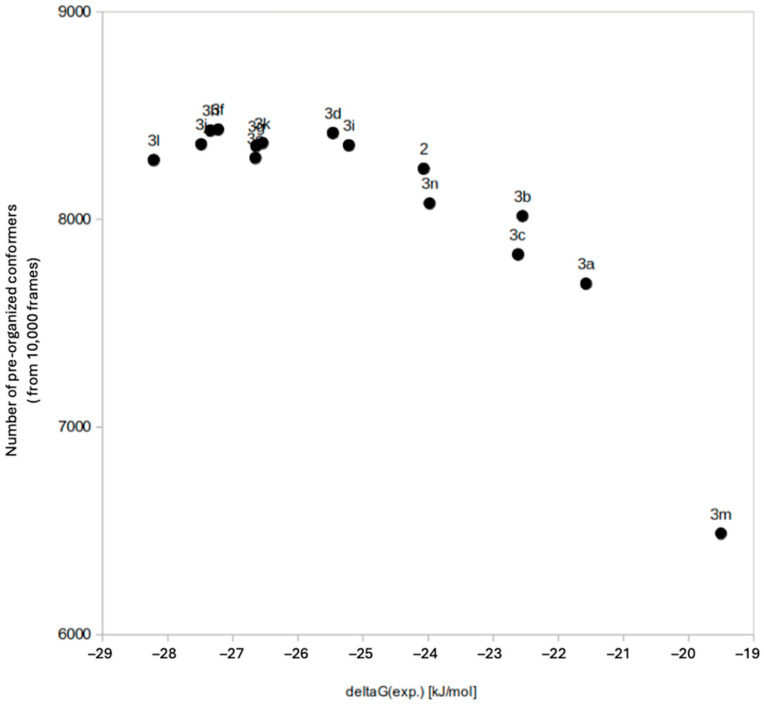
Degree of pre-organization shows trend with the binding affinity.

The *core conformation* parameter showed consistent distributions for all ligands, which is not unexpected because a remote isosteric replacement of the carboxylate has only a limited effect on the core conformation and stabilization. However, analysis of the parameter *acid orientation* revealed clear differences in the size and distribution of the ligands. To quantify these differences, we determined the portion of the conformations collected over an MD simulation period of 480 ns that would readily fit the binding site. More precisely, we calculated the root mean squared deviation (RMSD) of each MD conformation to the co-crystallized pose of lead compound **2** (PDB ID: 6EYI). Conformations with the RMSD value below 1.0 Å, indicating an excellent fit—or in other words a perfect pre-organization—were summed and divided by the total number of simulated conformations (10,000) (Figure 3).

For lead compound **2**, 82.4% of the MD conformations were pre-organized. Interestingly, all amide derivatives with improved affinity had a slightly higher proportion of pre-organized conformers by 83–84%. Weaker binding monosubstituted amides **3b** (80.2%), **3c** (78.3%), and hydroxamic acid derivative **3n** (80.8%) had fewer pre-organized conformers than lead **2**, followed by unsubstituted amide **3a** (76.9%). MD conformers of the weakest binder, tetrazole **3m** fitted to the binding site in only 64.9% of cases. A closer look at the non-fitting, non-pre-organized conformations revealed a tendency to form an unfavorable intramolecular stabilization between the carboxylic acid of **2** or its amide isosteres and the axial 4-OH of the galactose subunit. This stabilization, absent in the bound pose, was more frequent if there were more options for H-bond formation; e.g., unsubstituted amide **3a** can both donate to and accept H-bonds from Gal-4OH, increasing such undesired stabilizations even more frequently. The same applies for *N*-monoalkylated amides **3b** and **3c**, although the replacement of one N-H group with an alkyl substituent slightly decreases the proportion of undesired H-bonded conformations. The trend reverses with derivatives without H-bond donors, such as *N*,*N*-dimethylamide **3d**, pyrrolidine amide **3e** and all azetidine derivatives, having decreased odds of adopting pre-organized conformations, such as lead compound **2**. Interestingly, for all ligands, intramolecular H-bonding stabilization to equatorially oriented Gal-2OH was rare and did not account for the differences in pre-organization.

### 2.4. Protein–Ligand MD Simulations and Molecular Mechanics/Generalized Born Surface Area (MM-GB/SA) Analysis

To complement the insights obtained from the free ligand MD simulations, we also conducted five independent 48ns MD simulations for each of the 15 E-selectin ligands. Because it includes major simulation data, we took advantage of the automated MM-GB/SA scripts of the Schrodinger suite [27] in order to identify additional trends at the level of protein–ligand interactions contributing to differences in binding affinities. Overall, averaged MM-GB/SA binding free energies showed decent relative correlation with the experimental affinity data (R^2^ = 0.491, Supporting Information, Figure S4), which is a gratifying observation considering the narrow range of the experimental data spanning just 8.7 kJ/mol (2.1 kcal/mol) in free energy. The most prominent outlier is compound **2** with a carboxyl group, likely stemming from its pronounced electrostatic interaction (formal negative charge of −1), which is absent in amides. As expected, in absolute terms the MM-GB/SA binding free energies were overestimated. For highly charged systems, such as those involving Ca^2+^, the GB/SA approach is unreliable; Ca^2+^ self-energy and interaction terms can dominate the estimate, sometimes even producing positive (unfavorable) values. This limitation is well documented [28] but does not generally affect the relative trends. Separate analysis of H-bonding, lipophilic, and van der Waals components from the ΔΔG_binding_ MM-GB/SA outputs showed that the lipophilic free energy reached a slightly better correlation with the affinity (R^2^ = 0.558; Supporting Information, Figure S5) than the composite DG value. This indicates that substituents on the amide moiety may also engage in favorable lipophilic interactions with the E-selectin binding site, although to a very limited degree. In the bound pose, these substituents are oriented predominantly toward the bulk solvent, and the only nearby residue featuring a lipophilic character is the conformationally restricted Pro48 residue.

## 3. Conclusions

In this study, we rationalized the impact of carboxyl-to-amide isosteric replacements in a series of glycomimetic E-selectin ligands by combining crystallographic analysis, quantum chemical descriptors, conformational dynamics, and MM-GB/SA-based binding energy calculations. Despite the apparent loss of a canonical salt-bridge interaction upon removal of the carboxylate group, several amide analogs retained or even improved the binding affinity. Our results provide compelling evidence that such seemingly counterintuitive SAR trends can be rationalized by integrating subtle structural, electronic, and dynamic effects.

Crystallographic data confirmed consistent binding positions for all active ligands (see Supporting Information), with the conserved Le^x^-like trisaccharide scaffold fitting identically across the series. Although electron density maps did not allow for high-resolution discrimination of hydrogen-bonding geometries, the spatial orientation of amide carbonyls remained consistent with the key interactions with Arg97 and Tyr48.

Quantum chemical analyses ruled out a clear role for carbonyl oxygen partial charges or dipole moments in explaining the binding differences, especially given the complexity of competing local structural effects. Interestingly, a moderate correlation emerged between the amide nitrogen pyramidality and affinity, pointing toward a possible steric fit advantage of non-planar amides in the binding cavity. This underscores the value of geometric descriptors, which are often overlooked in favor of purely electronic parameters.

Molecular dynamics simulations of unbound ligands further revealed that improved binding was associated with higher degrees of solution-phase pre-organization toward the bound conformation. Notably, the azetidine amide and *N*,*N*-dialkyl amide derivatives showed fewer undesired intramolecular hydrogen bonds, which may impede protein binding. These findings suggest that fine-tuning the conformational landscape of the free ligand by limiting internal H-bond formation can promote binding-relevant poses and thereby enhance affinity.

Complementary MD simulations of ligand–protein complexes, paired with MM-GB/SA energy decomposition, supported these conclusions. Although the absolute energy values of the method were inflated owing to the presence of a calcium ion in the binding site, the relative trends showed satisfactory agreement with the experimental results. In particular, lipophilic interaction energies showed the highest correlation with experimental binding, indicating a minor yet measurable contribution of amide substituents to favorable protein contacts, likely with Pro48.

Taken together, our findings demonstrate that amide groups, traditionally viewed as inferior isosteres of carboxylates in a salt-bridge context, can maintain or even improve binding when embedded in an optimally pre-organized ligand scaffold and supported by a complementary protein microenvironment. Moreover, local geometry, such as amide nitrogen pyramidality and pre-organizational effects, emerged as key contributors to binding strength in this narrow SAR space.

These insights have broader implications for glycomimetic drug design: functional group replacements must be evaluated not only in isolation but also in the full context of ligand conformation, solvent interactions, and receptor flexibility. Future studies should explore further modifications of the amide scaffold, including constrained bicyclic systems or heteroatom substitutions, to enhance pre-organization or modulate solvation energetics.

Ultimately, this work illustrates how the detailed structural, electronic, and dynamic interrogation of a narrow SAR series can illuminate the underlying drivers of affinity, which can be generalized to the rational design of other ligand classes targeting lectins or similarly structured carbohydrate-binding proteins.

## 4. Materials and Methods

### 4.1. Crystallography

For co-crystallization and structure determination of E-selectin ligand complexes, see the Supporting Information.

### 4.2. Ab Initio Quantum Mechanical Calculations

All quantum mechanical calculations were performed using Gaussian 16 (Rev. C.01) software. Geometry optimization and harmonic frequency calculations were performed at the B3LYP-D3(BJ)/aug-cc-pVTZ level of theory. The B3LYP functional (https://doi.org/10.1063/1.464913) was augmented with Grimme’s D3 dispersion correction with Becke–Johnson damping (keyword EmpiricalDispersion = GD3BJ), which improves the description of noncovalent interactions in conformationally flexible and polar molecules (https://doi.org/10.1002/jcc.21759).

The aug-cc-pVTZ basis set (https://doi.org/10.1063/1.456153) was used for all atoms. This triple-ζ correlation-consistent basis set includes diffuse functions, which are essential for accurately modeling the structures, dipole moments, charge distributions, and solvation effects in neutral polar molecules such as *N*,*N*-disubstituted acetamides.

Geometries were optimized in the gas phase and in water, the latter using the SMD implicit solvation model (https://doi.org/10.1021/jp810292n) as implemented in Gaussian (SCRF = SMD). All geometry optimizations employed the Opt = Tight keyword to ensure a stricter convergence of the energy gradient and step size criteria. Harmonic frequency calculations (Freq = NoRaman) were used to verify that the optimized structures corresponded to true minima (no imaginary frequencies) and to obtain zero-point vibrational energy (ZPVE) and thermal corrections.

To improve the numerical accuracy of the energy gradients and properties derived from the electron density (e.g., dipole moments and electrostatic potentials), we used an Ultrafine DFT integration grid (Int = UltraFine). The desolvation free energy DG_s_ were calculated as the simple difference of free energy in the solvated and gas phases. All computed thermodynamic and solvation free energies were derived from the optimized structures and corrected using the thermal contributions at 298.15 K and 1 atm.

Natural Bond Orbital (NBO) analysis was performed using NBO 3.1, as integrated in Gaussian 16, via the Pop = (NBOread) keyword and specifying the control section ($NBO BNDIDX $END). This provided atomic natural charges, in particular the partial charge on the carbonyl oxygen, and Wiberg bond indices for key bonds, such as C=O and C–N. These quantities were used to assess electron delocalization and resonance effects across a congeneric series of amides.

### 4.3. Molecular Dynamics Simulations and Free Energy Calculations

All MD simulations were performed using the Desmond engine (version 2023-4) with the OPLS-2005 force field. The crystal structure of E-selectin in complex with a tetrasaccharide mimetic ligand (PDB ID: 4C16) was used as the starting point for protein–ligand simulations. The protein was prepared using Maestro’s Protein Preparation Wizard, including the addition of hydrogens, assignment of protonation states at pH 7.4 using PROPKA, and restrained minimization.

Ligands were generated using LigPrep, and simulations of the free ligands were conducted in cubic boxes of TIP3P water with a 15 Å buffer from the solute to the box edge. No salt was added beyond neutralizing the net charge with a single Na^+^ counterion for the acidic species. Each ligand system was equilibrated using the standard Desmond relaxation protocol followed by 480 ns of MD production in the NPT ensemble (300 K, 1 atm). Trajectory snapshots were saved every 48 ps, yielding 10,000 frames per ligand.

Simulations of the ligand–protein complexes were set up similarly, but embedded in orthorhombic TIP3P water boxes with a 10 Å buffer. Again, no additional salt was added beyond the counterions for net neutralization. Each complex was simulated for 48 ns using the same ensemble settings and Desmond default relaxation protocol. For each ligand–protein complex, quintuplicate simulations were initialized using a different random number seed.

Long-range electrostatics were treated using the particle mesh Ewald method; the RESPA integrator was used with a 2.0 fs time step. Temperature and pressure controls were maintained using the Nosé–Hoover thermostat and the Martyna–Tobias–Klein barostat.

Binding free energies were estimated via the Molecular Mechanics Generalized Born Surface Area (MM-GB/SA) method using the Prime module (Schrödinger). For each complex, frames 10–110 were extracted from the trajectory for the MM-GB/SA analysis, as longer simulations are not advised for this method because of configurational averaging artifacts. If not stated otherwise, default protocols were used.

## Figures and Tables

**Table 1:** Sialyl Lewis^x^ (**1**), the glycomimetic parent compound **2** and amide glycomimetics **3a-n** and their *K*_D_ s measured by microscale thermophoresis (MST). Syntheses and affinity data are reported in Ref. [25] For co-crystallization and structure determination of E-Selectin ligand complexes see Supporting Information.

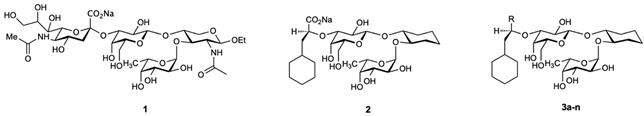							
Cpd.	R	Affinity *K_D_* MST [µM] (95% CI)	PDB ID	Cpd.	R	Affinity *K_D_* MST [µM] (95% CI)	PDB ID
**1**	-	873 (750–1024)	4CSY	**3g**	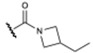	21.5 (16.7–27.7)	9HGY
**2**	-	60.7 (52.2–70.6)	6EYI	**3h**	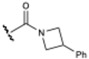	16.2 (12.9–20.3)	-
**3a**	C(=O)NH_2_	166 (145–189)	-	**3i**	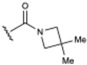	38.2 (32.4–45.7)	-
**3b**	C(=O)NHMe	112 (98.6–128)	-	**3j**	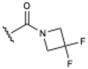	15.3 (13.1–17.9)	-
**3c**	C(=O)NHEt	109 (93.5–127)	-	**3k**	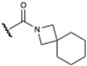	22.4 (17.2–29.0)	9HGX
**3d**	C(=O)NMe_2_	34.6 (30.2–39.5)	9HGU	**3l**	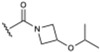	11.4 (9.5–13.6)	-
**3e**	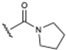	21.4 (17.7–25.8)	9HGV	**3m**	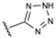	383 (350–416)	-
**3f**	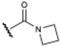	17.0 (14.9–19.3)	9HGW	**3n**	C(=O)NHOH	63.0 (62.7–95.1)	-

**Table 2:** Molecular properties of small amide model systems calculated using quantum mechanics methods in gas and solvent phase. The R^2^ coefficient indicates how different properties correlate with ΔG_exp_. (BO: bond order, Q: net charge, χ: pyramidality, µ: dipole moment, ΔG_solv_. _(calc.)_: calculated free energy of solvation).

		Gas-Phase Properties				Solvent-Phase Properties (Water)			
	DG_bind. (exp.)_	BO_C=O_	Q_O_	χ_N_	μ	Q_O_	χ_N_	μ	DG_solv.(calc.)_
Cpd.	[kJ/mol]		[e^−^]	[°]	[Debye]	[e^−^]	[°]	[Debye]	[kcal/mol]
**3l**	−28.21	1.659	−0.621	15.3	4.1	−0.758	15.3	6.3	−10.4
**3j**	−27.48	1.652	−0.608	15.7	2.9	−0.734	18.9	4.7	−9.4
**3h**	−27.34	1.656	−0.622	12.2	4.2	−0.765	7.1	6.7	−10.4
**3f**	−27.22	1.654	−0.625	11.8	4.4	−0.763	15	6.9	−9.0
**3e**	−26.65	1.644	−0.635	0.8	4.4	−0.770	0.2	7.0	−8.9
**3g**	−26.64	1.653	−0.625	12.8	4.5	−0.767	12.8	7.1	−8.5
**3k**	−26.54	1.649	−0.627	8.4	4.6	−0.765	14.7	7.1	−8.8
**3d**	−25.46	1.649	−0.634	0	3.9	−0.758	9	6.4	−7.1
**3i**	−25.22	1.652	−0.625	9.9	4.5	−0.764	14.1	7.0	−8.3
**3c**	−22.62	1.660	−0.635	3.7	3.7	−0.763	0	6.2	−9.2
**3b**	−22.55	1.664	−0.630	0	3.9	−0.762	0	6.2	−8.4
**3a**	−21.57	1.692	−0.616	0	3.9	−0.751	0	6.0	−8.6
**R^2^ =**	n.a.	0.468	0.059	0.569	0.033	0.000	0.553	0.027	0.192

## Data Availability

The original contributions presented in this study are included in the article. Further inquiries can be directed to the corresponding author.

## Supplementary Materials

The following supporting information can be downloaded at: https://www.mdpi.com/article/10.3390/molecules30244751/s1. Figure S1. Superposition of the crystal poses of lead compound **2** and azetidine amide derivative **3f**; Figure S2. 3D ab initio optimized geometries of small model systems in water; Figure S3. Correlation of amide nitrogen pyramidality with the experimental binding free energy (only amides); Figure S4. Correlation of the total MM-GB/SA binding free energy with the experimental binding free energy. Figure S5. Correlation of the lipophilic component of the MM-GB/SA binding free energy with the experimental binding free energy; Co-crystallization and Structure Determination of E-Selectin ligand complexes; Isothermal Titration Calorimetry (ITC); Figure S6. Close-up views of the ligand interactions in co-crystal structures of E-selectin with 3d (A, PDB 9HGU), 3e (B, PDB 9HGV), 3f (C, PDB 9HGW), 3k (D, PDB 9HGX), and 3g (E, PDB 9HGY). Ligandinteracting amino acids are labeled; the Ca^2+^ ion is shown as a green sphere. Oxygen and nitrogen atoms are colored red and blue, respectively; Figure S7. ITC thermograms and binding isotherms of compound 2 (A) and 3f (B); Table S1. Data collection and refinement statistics of E-selectin ligand complexes. References [10,29,30,31,32,33,34,35,36,37,38,39,40,41].

## Abbreviations

The following abbreviations are used in this manuscript:

MST	Microscale thermophoresis
MD	Molecular dynamics
ITC	Isothermal titration calorimetry
PDB	Protein data base
NBO	Natural bond orbital
BO	Bond order
Q	Net charge
RMSD	Root mean squared deviation
MM-GB/SA	Molecular Mechanics/Generalized Born Surface Area
SMD	Implicit solvent model
OPLS	Optimized Potentials for Liquid Simulations
TIP3P	Transferable Intermolecular Potential 3-Point
