# Supplementary material for: Acid Versus Amide—Facts and Fallacies: A Case Study in Glycomimetic Ligand Design
Source: Molecules. 2025 Dec 12;30(24):4751. doi: 10.3390/molecules30244751 (PMC12736226; doi:10.3390/molecules30244751)
Supplement: Supplementary file 1 [file molecules-30-04751-s001.zip › molecules-3979648-supplementary.pdf]

Supporting Information

Acid vs Amide - Facts and Fallacies

Martin Smieško[1], Roman P. Jakob[2], Tobias Mühlethaler[3], Roland C. Preston[3], Timm Maier,[2] Beat Ernst[3]*

[1]Computational Pharmacy, Department of Pharmaceutical Sciences, University of Basel, Klingelbergstrasse 50, 4056 Basel, Switzerland

[2] Department Biozentrum, Structural Area Focal Biology, University of Basel, Spitalstrasse 41, 4056 Basel,Switzerland

[3]Molecular Pharmacy, Department of Pharmaceutical Sciences, University of Basel Klingelbergstrasse 50, 4056 Basel, Switzerland

*corresponding author

Content

1. Figures S1 – S5
2. Co-crystallization and Structure Determination of E-Selectin ligand complexes
3. Isothermal Titration Calorimetry

1. Figures S1 – S5

Figure S1. Superposition of crystal poses of the lead compound **2** (green carbons) and azetidine amide derivative **3f** (orange carbons).

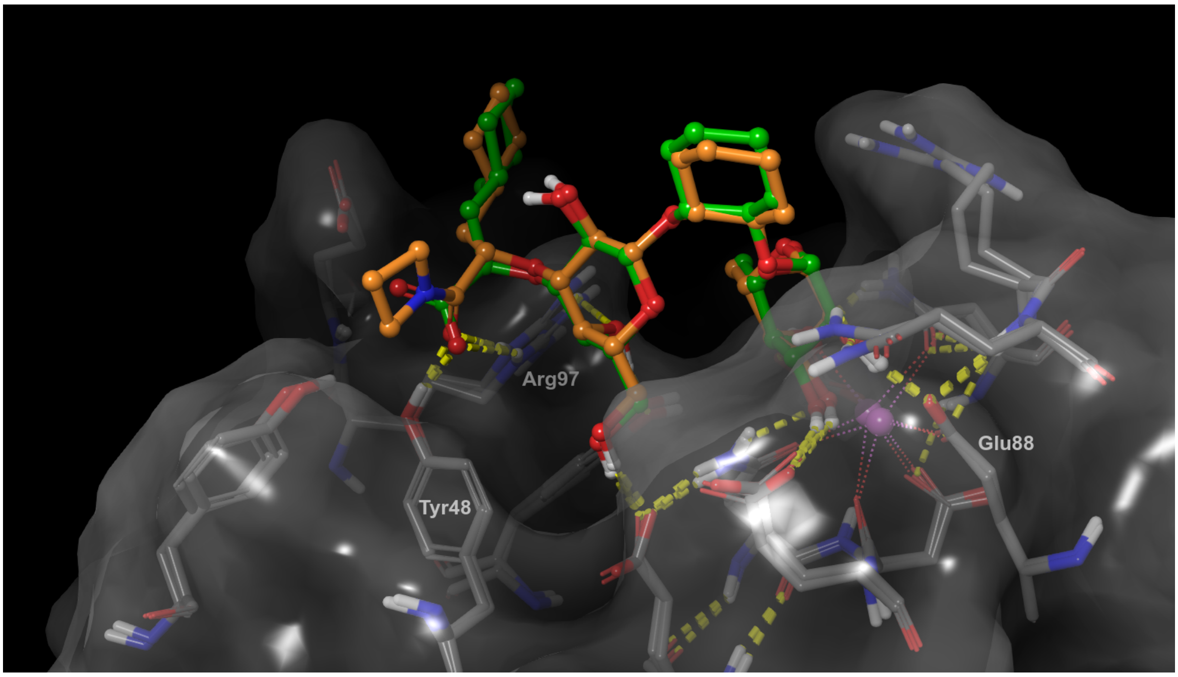

Figure S2. 3D *ab initio* optimized geometries of small model systems in water.

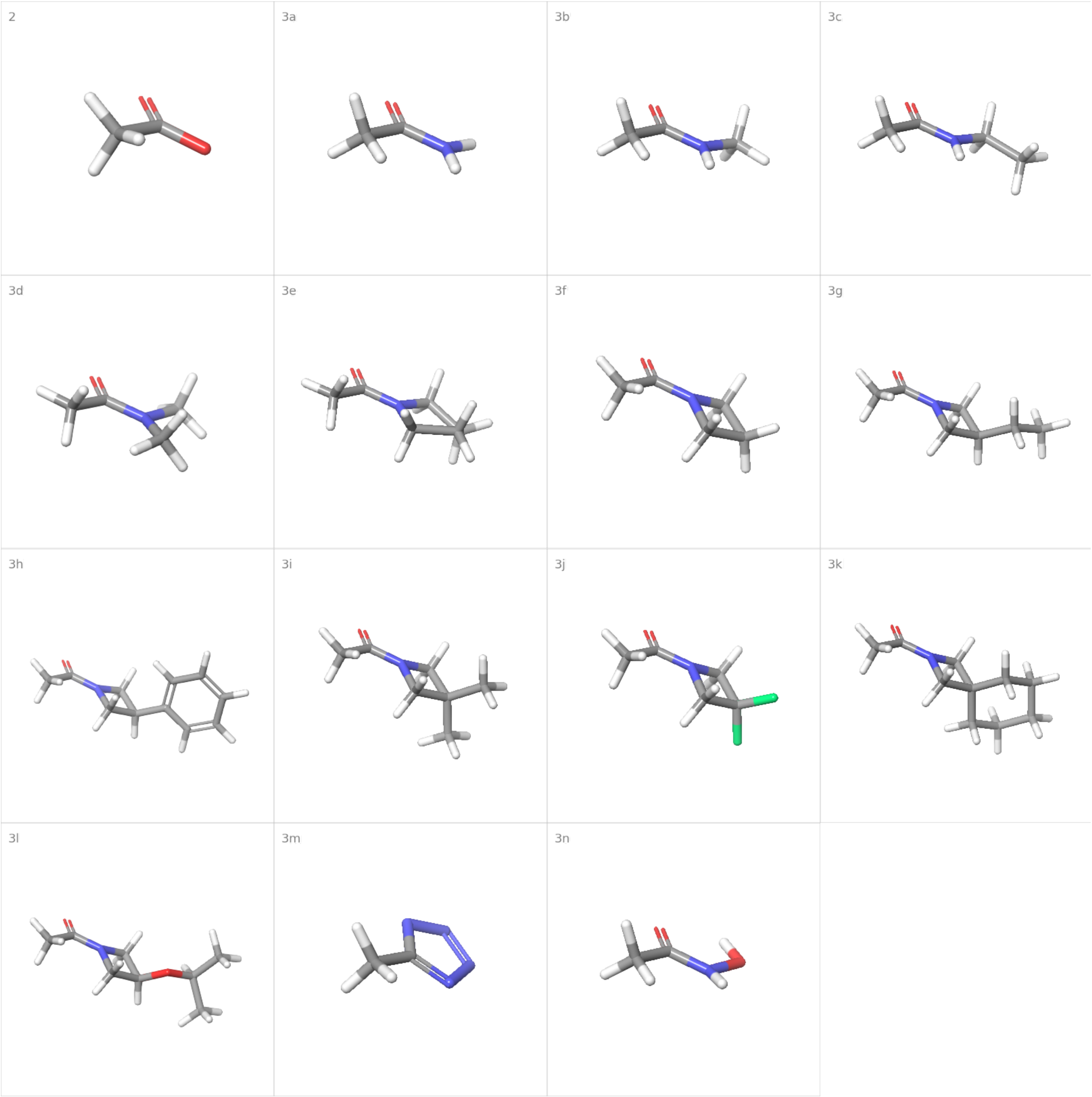

Figure S3. Correlation of amide nitrogen pyramidality to the experimental binding free energy (only amides).

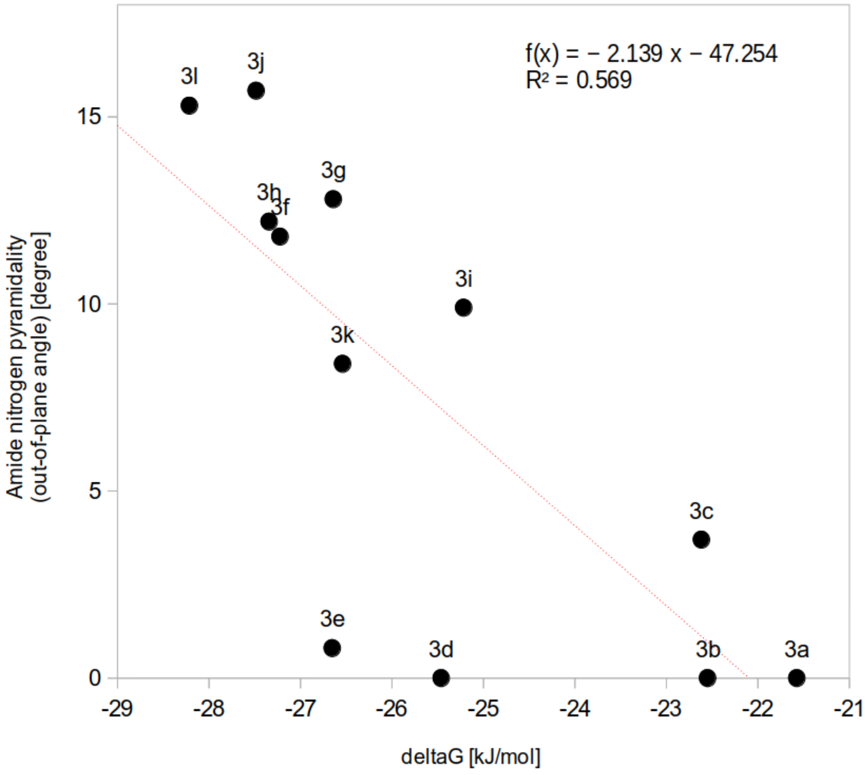

Figure S4. Correlation of the total MM-GB/SA binding free energy to the experimental binding free energy (all compounds).

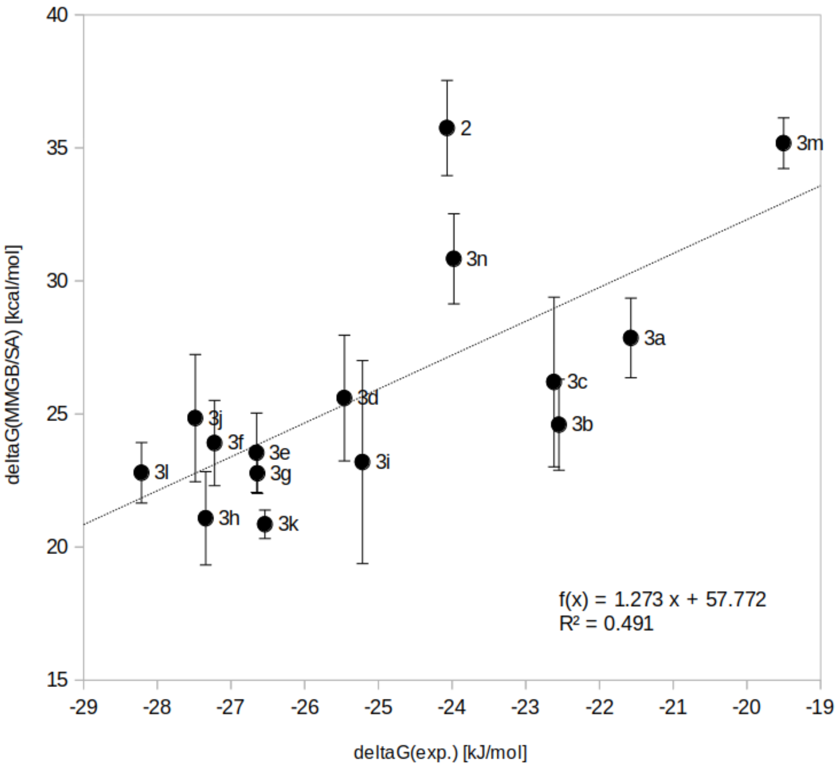

Figure S5. Correlation of the lipophilic component of the MM-GB/SA binding free energy to the experimental binding free energy (all compounds).

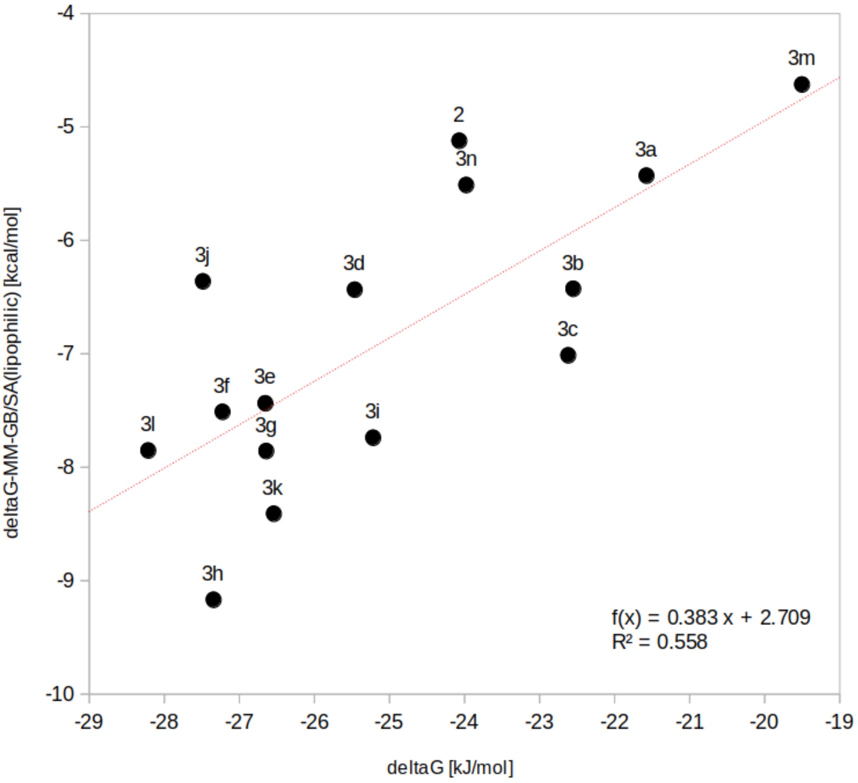

2. Co-crystallization and Structure Determination of E-Selectin ligand complexes

Deglycosylated E-selectin[S6] was co-crystallized with ligands using a sitting drop method in the presence of 0.2 M $CaCl_2$, 0.1 M 3-(N-morpholino) propanesulfonic acid (MOPS) at pH 6.2, and 20% polyethylene glycol 8,000 as the precipitant. Crystals were optimized by seeding into a solution containing 0.2 M $CaCl_2$, 0.1 M MOPS (pH 6.2), and 12–14% polyethylene glycol 8000. Large, plate-like crystals formed and reached their final size within one week after seeding. Prior to flash-freezing in liquid nitrogen, the crystallization drops were coated with perfluoropolyether (Hampton Research, CA). Diffraction data were collected at the PXI (X06SA) beamline of the Swiss Light Source, Paul Scherrer Institute, Villigen, Switzerland, and processed using XDS software[S1] and scaled using XSCALE[S2] (Kabsch 2010) or AIMLESS.[S3]

The structures of the E-selectin ligand complexes were determined by molecular replacement using Phaser[S4] within the CCP4 software package[S5] using the E-selectin/sialyl Lewis^x structure (PDB code 4CSY)[S6] as search model. Refinement was performed with the BUSTER software[S7] and PHENIX[S8]. Coot[S9] was used for model building. Ligand restraints were generated using PRODRG[S10] and eLBOW.[S11] Molprobity was used for validation.[S12] Data collection and refinement statistics are listed in Table S1.

Figures were generated with PyMol.[S13] The data have been deposited under PDB accession codes 9HGU, 9HGV, 9HGW, 9HGX, and 9HGY.

Table S1. Data collection and refinement statistics of E-selectin ligand complexes.

	E-selectin/ **3d**	E-selectin/ **3e**	E-selectin/ **3f**	E-selectin/ **3k**	E-selectin/ **3g**
PDB ID	9HGU	9HGV	9HGW	9HGX	9HGY
Wavelength (Å)	0.99999	0.99999	0.99999	0.99997	1.00003
Resolution range (Å)	57.2 – 2.30 (2.40 – 2.30)*	46.3 – 2.46 (2.71 – 2.46)*	46.4 – 2.20 (2.34 – 2.20)*	57.5 – 2.75 (3.15 – 2.75)*	57.3 – 2.50 (2.75 – 2.50)*
Space group	C 1 2 1	C 1 2 1	C 1 2 1	C 1 2 1	C 1 2 1
Unit cell	92.7 72.8 52.3	92.8 73.0 52.4	92.9 72.9 52.3	93.0 73.2 52.4	93.1 72.9 52.3
α, β, γ (°)	90 94.1 90	90 94.1 90	90 94.3 90	90 93.6 90	90 94.3 90
Total reflections	62,798 (7,654)	56,694 (14,134)	145,296 (6,787)	55,394 (17,680)	82,546 (20,867)
Unique reflections	29,145 (3,516)	24,450 (6,089)	17,460 (2,789)	17,841 (5,892)	23,394 (5,774)
Multiplicity	2.2 (2.2)	2.3 (2.3)	4.1 (2.3)	3.1 (3.0)	3.5 (3.6)
Completeness (%)	96.8 (95.7)	98.7 (98.6)	98.3 (95.5)	99.7 (99.3)	98.1 (97.1)
Mean I/sigma(I)	7.5 (0.8)	7.4 (0.9)	8.2 (1.2)	5.8 (1.2)	9.2 (1.3)
Wilson B-factor	60.9	65.5	48.1	60.0	58.4
R-merge	0.058 (1.07)	0.066 (0.99)	0.223 (1.21)	0.152 (0.81)	0.086 (0.96)
R-pim	0.049 (0.89)	0.053 (0.78)	0.092 (0.61)	0.109 (0.66)	0.054 (0.58)
CC1/2	0.998 (0.53)	0.997 (0.55)	0.985 (0.54)	0.994 (0.67)	0.999 (0.69)
Reflections used in Refinement	15,051 (1,848)	12,603 (3,116)	17,460 (2,789)	9,176 (3,012)	11,958 (2,934)
R-work	0.231 (0.363)	0.236 (0.382)	0.235 (0.378)	0.225 (0.318)	0.229 (0.347)
R-free	0.269 (0.403)	0.271 (0.425)	0.248 (0.438)	0.270 (0.349)	0.272 (0.382)
Number of non-hydrogen atoms	2,340	2,323	2,360	2,400	2,389
macromolecules	2,176	2,176	2,179	2,176	2,76
ligands	142	144	143	148	145
water	22	3	38	76	68
Protein residues	280	280	280	280	280
RMS(bonds)	0.004	0.006	0.003	0.014	0.014
RMS(angles)	0.75	1.01	0.75	1.85	1.83
Ramachandran favored (%)	96.0	96.4	97.1	96.4	95.3
Ramachandran outliers (%)	0.4	0.4	0.7	0.7	0.7
Rotamer outliers (%)	0.4	0.4	0.8	1.2	1.4
Clashscore	5.9	5.2	3.9	2.5	2.2
B-factor (All)	80.3	82.8	57.2	77.0	73.0

RMS indicates root mean square. Single crystals were used.
*Values in parentheses are for highest-resolution shell.

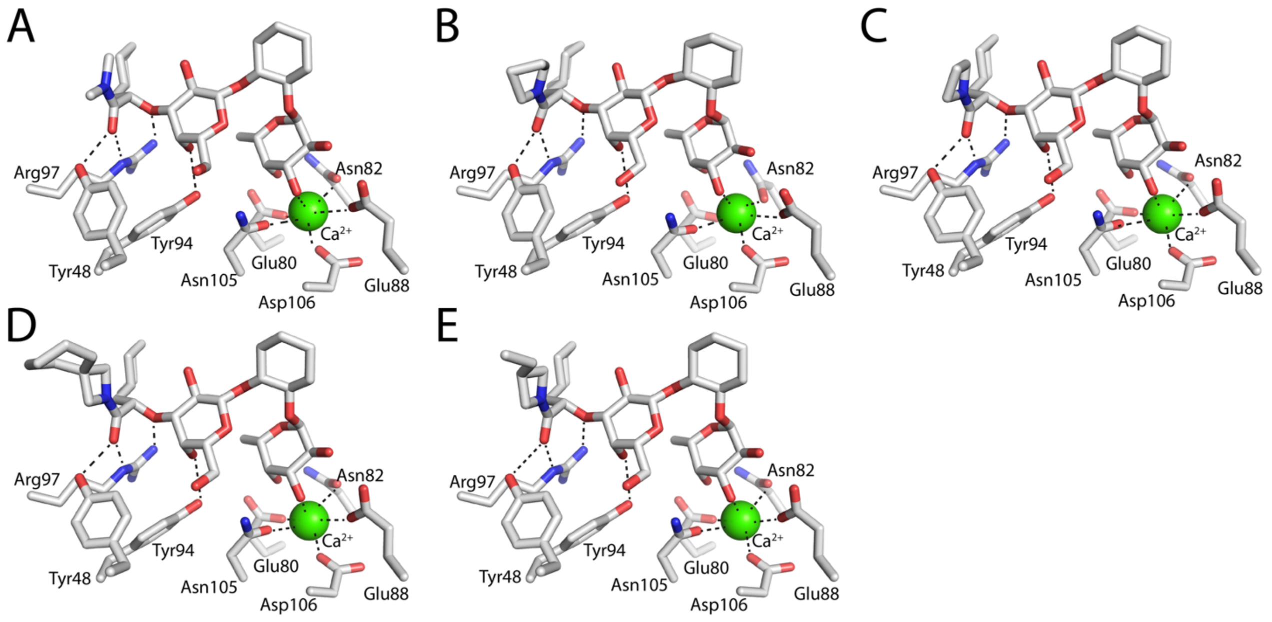

Figure S6. Close-up views of the ligand interactions in co-crystal structures of E-selectin with **3d** (**A**, PDB 9HGU), **3e** (**B**, PDB 9HGV), **3f** (**C**, PDB 9HGW), **3k** (**D**, PDB 9HGX), and **3g** (**E**, PDB 9HGY). Ligand-interacting amino acids are labeled; the Ca^{2+} ion is shown as a green sphere. Oxygen and nitrogen atoms are colored red and blue, respectively.

3. Isothermal Titration Calorimetry

ITC data were collected with a MicroCal VP-ITC (Malvern Panalytical) at 25 °C, 10 µcal/sec reference power, 307 r.p.m. stirring speed, and high gain. 18 injections of 15 µl of 2 mM **2** were titrated at 300 sec intervals into 90.9 µM E-selectin; or 54 injections of 6 µl of 2 mM **3f** were titrated at 450 sec intervals into 106 µM E-selectin. Baseline adjustment, peak integration, and figure preparation were carried out using Origin 7.0 (OriginLab, Northampton, MA, USA). The data was fitted using a 1:1 hetero-association model enabling correction for inactive protein for **3f** or with the *N*-value fixed to 1 for **2** with SEDPHAT (version 12.1b)[S14]

Comp.	K_D [µM]	ΔG_{obs} [kJ mol^{-1}]	ΔH_{obs} [kJ mol^{-1}]	$-T\Delta S_{obs}$ [kJ mol^{-1}]
**2**	60.7 (43.4 – 84.8)	-24.1	-5.3 (-4.5 – -6.5)	-18.8
**3f**	23.6 (21.5 – 25.9)	-26.4	-6.7 (-6.5 – -7.0)	-19.7

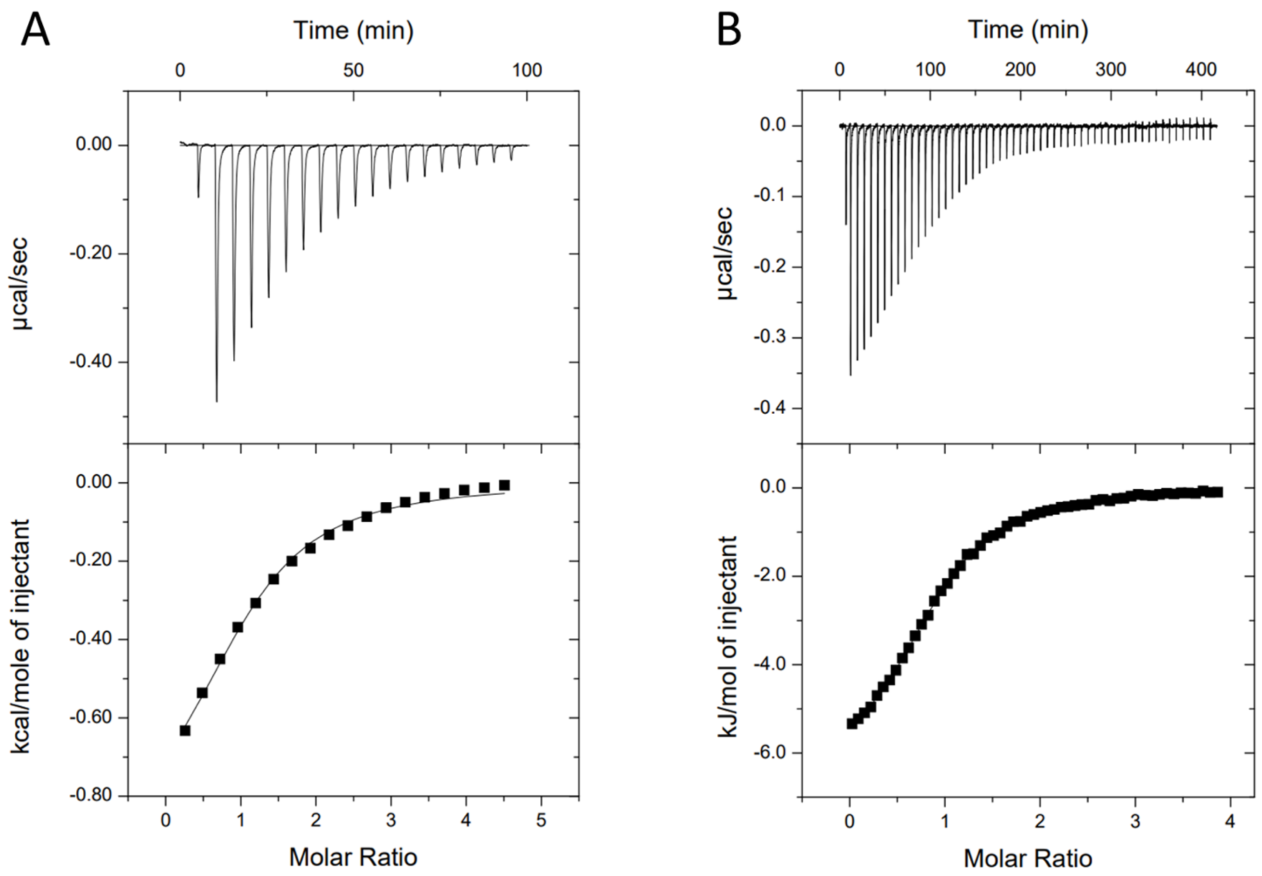

Figure S7. ITC thermograms and binding isotherms of compound **2** (A) and **3f** (B).

References

[S1] Kabsch, W. Xds. *Acta Crystallogr. D Biol. Crystallogr.* **2012**, *66(Pt 2)*, 125-132.

[S2] Kabsch, W. Integration, scaling, space-group assignment and post-refinement. *Acta Crystallogr. D Biol. Crystallogr.* **2010**, *66(Pt 2)*, 133-144.

[S3] Evans, P. R.; Murshudov, G. N. How good are my data and what is the resolution? *Acta Crystallogr. D Biol. Crystallogr.* **2013**, *69(Pt 7)*, 1204-1214.

[S4] McCoy, A. J.; Grosse-Kunstleve, R. W.; Adams, P. D.; Winn, M. D.; Storoni, L. C.; Read, R. J. Phaser crystallographic software. *J. Appl. Crystallogr.* **2007**, *40(Pt 4)*, 658-674.

[S5] Winn, M. D.; Ballard, C. C.; Cowtan, K. D.; Dodson, E. J.; Emsley, P.; Evans, P. R.; Keegan, R. M.; Krissinel, E. B.; Leslie, A. G.; McCoy, A.; McNicholas, S. J.; Murshudov, G. N.; Pannu, N. S.; Potterton, E. A.; Powell, H. R.; Read, R. J.; Vagin, A.; Wilson, K. S. Overview of the CCP4 suite and current developments. *Acta. Crystallogr. D Biol. Crystallogr.* **2011**, *67(Pt 4)*, 235-242.

[S6] Preston, R. C.; Jakob, R. P.; Binder, F. P.; Sager, C. P.; Ernst, B.; Maier, T. E-selectin ligand complexes adopt an extended high-affinity conformation. *J. Mol. Cell Biol.* **2016**, *8*, 62-72.

[S7] Blanc, E.; Roversi, P.; Vonrhein, C.; Flensburg, C.; Lea, S. M.; Bricogne, G. Refinement of severely incomplete structures with maximum likelihood in BUSTER-TNT. *Acta Crystallogr. D Biol. Crystallogr.* **2004**, *60(Pt 12 Pt 1)*, 2210-2221.

[S8] Adams, P. D.; Gopal, K.; Grosse-Kunstleve, R. W.; Hung, L. W.; Ioerger, T. R.; McCoy, A. J.; Moriarty, N. W.; Pai, R. K.; Read, R. J.; Romo, T. D.; Sacchettini, J. C.; Sauter, N. K.; Storoni L. C.; Terwilliger T. C. Recent developments in the PHENIX software for automated crystallographic structure determination. *J. Synchrotron Radiat.* **2004**, *11(Pt 1)*, 53-55.

[S9] Emsley, P.; Cowtan, K. Coot: model-building tools for molecular graphics. *Acta Crystallogr. D Biol. Crystallogr.* **2004**, *60(Pt 12 Pt 1)*, 2126-2132.

[S10] Schuttelkopf, A. W.; van Aalten, D. M. PRODRG: a tool for high-throughput crystallography of protein-ligand complexes. *Acta Crystallogr. D Biol. Crystallogr.* **2004**, *60(Pt 8)*, 1355-1363.

[S11] Moriarty, N. W., Grosse-Kunstleve, R. W.; Adams P. D. Electronic Ligand Builder and Optimization Workbench (eLBOW): a tool for ligand coordinate and restraint generation. *Acta Crystallogr. D Biol. Crystallogr.* **2009**, *65(Pt 10)*, 1074-1080.

[S12] Chen, V. B., Arendall, 3rd, W. B.; Headd, J. J.; Keedy, D. A.; Immormino, R. M.; Kapral, G. J.; Murray, L. W.; Richardson, J. S.; Richardson, D. C. MolProbity: all-atom structure validation for macromolecular crystallography. *Acta Crystallogr D Biol Crystallogr* **2010**, *66(Pt 1)*, 12-21.

[S13] Schrodinger, L. L. C. The PyMOL Molecular Graphics System, **2015**, Version 1.8.

[S14] Zhao, H., Piszczek, G. Schuck, P. SEDPHAT–a platform for global ITC analysis and global multi-method analysis of molecular interactions. *Methods* **2015**, *76*, 137–148.
